# Fatigue at baseline is associated with geriatric impairments and represents an adverse prognostic factor in older patients with a hematological malignancy

**DOI:** 10.1007/s00277-018-3420-8

**Published:** 2018-07-28

**Authors:** F. Hofer, K. A. Koinig, L. Nagl, B. Borjan, R. Stauder

**Affiliations:** Department of Internal Medicine V (Hematology and Oncology), Innsbruck Medical University, Anichstrasse 35, 6020 Innsbruck, Austria

**Keywords:** Quality of life, Fatigue, Geriatric assessment, Hematological malignancies, Patient-reported outcomes

## Abstract

Prospective data on fatigue in elderly persons with a hematological malignancy are rare. We aimed to determine the prevalence of fatigue and its association with clinical outcome and geriatric impairments in older individuals newly diagnosed with blood cancer. The EORTC QLQ-C30 and a multidimensional geriatric assessment (MGA) were performed in parallel in 149 consecutive patients aged > 67 years (median 77.8 years) at Innsbruck Medical University between January 2009 and April 2016. Fatigue as defined by EORTC QLQ-C30 was the most prevalent symptom (84%) and was significantly associated with self-reported role and physical functioning, global health status and insomnia, dyspnea, and loss of appetite (*p* < 0.001). Remarkably, pronounced fatigue was associated with impaired performance status and objective functional capacities in MGA, with altered depression scoring, G8 screening, and elevation of serum inflammation markers (*p* < 0.001). Patients with minor fatigue had a median overall survival (OS) of 26.4 months, whereas those with marked fatigue displayed an OS of 7.0 months (*p* < 0.001). The association between fatigue and shortened OS was supported in multivariate analyses (HR 1.74, CI 1.09–2.76; *p* = 0.021). Fatigue is seen to have a high prevalence and to be an adverse prognostic factor in elderly patients with a hematological malignancy. The strong impact of fatigue on clinical performance and OS emphasizes the relevance of patient-reported outcomes in individualized treatment algorithms. Patients will benefit from identification of fatigue, allowing timely interventions. The correlation between fatigue, impaired performance, nutritional status, and inflammation might suggest an underlying common pathway.

## Introduction

Hematological malignancies are typical diseases of elderly with a median age at diagnosis of > 70 years for the most common subtypes such as chronic lymphocytic leukemia (CLL), diffuse large B cell lymphoma (DLBCL), multiple myeloma, acute myeloid leukemia (AML) or myelodysplastic syndromes (MDS) [1–3]. Thus, 60% of patients with malignant hemopathies are today older than 65 years and this proportion will continue to increase in the future [4, 5]. Based on dramatic demographic changes in Western communities as well as in developing countries, the proportion of individuals ≥ 65 years will rise to 28% and of those who are ≥ 80 years will rise to 12% by the year 2060 [5]. Thus, a continuous increase in the number of elderly patients suffering from a hematological malignancy has to be foreseen in the next years [4].

The clinical course in elderly patients is often characterized by a high symptom burden, with self-reported fatigue being one of the most troublesome symptoms [6–8]. The symptom complex of fatigue is described as tiredness without benefit from sleep, impaired physical performance capacity, lack of energy, helplessness, and depressed mood [6]. In the last years, fatigue and health-related quality of life (HRQoL) have been identified as typical patient-reported outcomes (PROs). PROs have been introduced in clinical trials and in daily practice to integrate the patient’s perspective [9]. The EORTC Quality of Life Questionnaire Core 30 (EORTC QLQ-C30) represents a typical score for assessing PROs, such as symptoms and the subjective health status of patients [10, 11]. So far, data on the prevalence and the impact of fatigue on clinical presentation and outcome in hematological malignancies in older patients are rare.

Evaluation of elderly patients with hematological malignancies is complicated by the aging process, namely the presence of comorbidities, malnutrition, and impairments in functional capacities [4, 12]. Multidimensional geriatric assessment (MGA) was developed to evaluate social, emotional, cognitive, physical, and nutritional aspects of patients’ health status, as well as comorbidities and patients’ symptom burden. It is recommended that MGA be integrated as a parameter in decision making, in order to form the basis for interventions and evaluate the supportive care needs of older persons with cancer [13].

Goal of this study was to analyze (a) the prevalence of fatigue as assessed by EORTC QLQ-C30, (b) the association between fatigue and geriatric impairments, and (c) the prognostic relevance of fatigue in a cohort of elderly patients with a hematological malignancy at initial diagnosis.

## Methods

### Patients

Between January 2009 and April 2016, patients newly diagnosed with a hematological malignancy at Innsbruck Medical University Hospital in the 70th year of life or older were included in this study. Thus, in 156 persons, an EORTC QLQ-C30 and a multidimensional geriatric assessment were performed in parallel at initial diagnosis. Of the four persons in the < 70 years category, three were in the 70th year of life. One patient aged 67 years was erroneously included. Since age did not significantly correlate with overall survival in univariate analysis, this patient remained in the analysis. Hematological malignancies were classified as described recently [14]. Exclusion criteria were presence of an additional tumor at time of assessment and lacking ability to obtain patient’s informed consent.

### Patient characteristics and geriatric assessment

The EORTC QLQ-C30 addresses different items of patient’s QoL: patient’s global health status, five functional scales, and nine symptom scales [15]. Fatigue is addressed with the three questions “Did you need to rest?”, “Have you felt weak?”, “Were you tired?” Possible answers are: 1—not at all; 2—a little; 3—quite a bit; and 4—very much. The prevalence of symptoms and impairments was classified in three categories: not at all (0), mild (1–66), and moderate to severe (67–100) [6, 16]. To analyze the impact of fatigue on clinical outcome, fatigue was divided in two categories as recently suggested by Efficace et al.: no or mild versus strong fatigue with a cut-off at 45 on the EORTC QLQ-C30 scale (ranging from 0 = no symptom to 100 = strong symptom) [17]. The MGA included 13 questionnaires assessing performance status (WHO, Karnofsky), comorbidities (CCI, HCT-CI, CIRS-G), nutritional status (MNA), mental state (MMSE, GDS), social support (FSozu), functional capacities (IADL, ADL, TUG), and the screening tool G8. In addition, demographic data and laboratory parameters (serum ferritin, transferrin saturation, C-reactive protein (CRP), serum albumin, fibrinogen) were included [14]. This study was approved by the local ethics committee. Written informed consent was obtained from all patients.

### Statistical analyses

SPSS (IBM Corporation, Armonk, NY, USA) was used for all statistical analyses. For comparison of fatigue with parameters of QoL and laboratory values, we used Spearman’s rank correlations. Four-year overall survival was analyzed with Kaplan-Meier methods and the log rank test. Hazard ratios were first analyzed in a univariate Cox proportional hazard regression. Parameters statistically significant in the univariate analyses were then included in the multivariate Cox regressions, additionally assessing the significance of the parameters with a bootstrapped model [18]. In multivariate analyses, we avoided auto-correlated indices and used CCI as comorbidity score and IADL as activity score. Four patients without ferritin measurements were excluded from the multivariate analyses (*n* = 145).

## Results

### Patient characteristics

MGA and EORTC QLQ-C30 were performed in 156 patients. Three patients were excluded from analysis because of an additional solid tumor; one patient was lost to follow-up, and in one other patient, the diagnosis of a hematological malignancy was not definitively confirmed. Two patients were excluded due to non-completion of more than three questionnaires. Finally, 149 patients were included in the analysis. Age at inclusion varied from 67.1 to 95.3 years with a median age of 77.8 years. At time of analysis (cut-off April 2017), 34 patients were still alive, while 115 patients were deceased (Table 1). Median OS was 13.0 months.

**Table 1:** Patient characteristics at baseline

	Total (*N* = 149)	No/mild fatigue < 45 (*N* = 82)	Strong fatigue > 45 (*N* = 67)	*P* value
Sex				ns
Male	80 (54%)	43	37	
Female	69 (46%)	39	30	
Age				ns
< 70 years	4 (3%)	1	3	
70–79 years	87 (58%)	45	42	
80–89 years	53 (36%)	33	20	
> 90 years	5 (3%)	3	2	
Entities				ns
Myelodysplastic syndromes	43 (29%)	29	14	
Acute myeloid leukemia	48 (32%)	22	26	
Myeloproliferative neoplasms^a^	10 (7%)	5	5	
Non-Hodgkin’s lymphoma—indolent^b^	13 (9%)	7	6	
Non-Hodgkin’s lymphoma—aggressive^c^	33 (22%)	19	14	
Multiple myeloma	2 (1%)	0	2	
Geriatric impairments in MGA				0.001
</= 3	95 (64%)	65	30	
> 3	54 (36%)	17	37	
Survival				0.014
Alive	34 (29%)	25	9	
Dead	115 (71%)	57	58	
Serum ferritin	*N* = 145			ns
< 400 μg/l	79 (54%)	47	32	
> 400 μg/l	66 (46%)	35	31	

^a^Myeloproliferative neoplasms included chronic myelomonocytic leukemia, chronic myeloid leukemia, and myelodysplastic syndromes—myeloproliferative neoplasm unclassifiable

^b^Non-Hodgkin’s lymphoma-indolent included B cell chronic lymphocytic leukemia, small lymphocytic non-Hodgkin’s lymphoma, Morbus Waldenström, low-grade mucosa-associated lymphoid tissue non-Hodgkin’s lymphoma, and hairy cell leukemia

^c^Non-Hodgkin’s lymphoma-aggressive included diffuse large B cell lymphoma, mantle cell lymphoma, prolymphocytic leukemia, and T cell non-Hodgkin’s lymphoma

*MGA* multidimensional geriatric assessment, *ns* not significant; *P* values < 0.05 were considered significant; *P* values were calculated using Chi-square tests

### Prevalence of symptoms and impairments

**Fig. 1:**
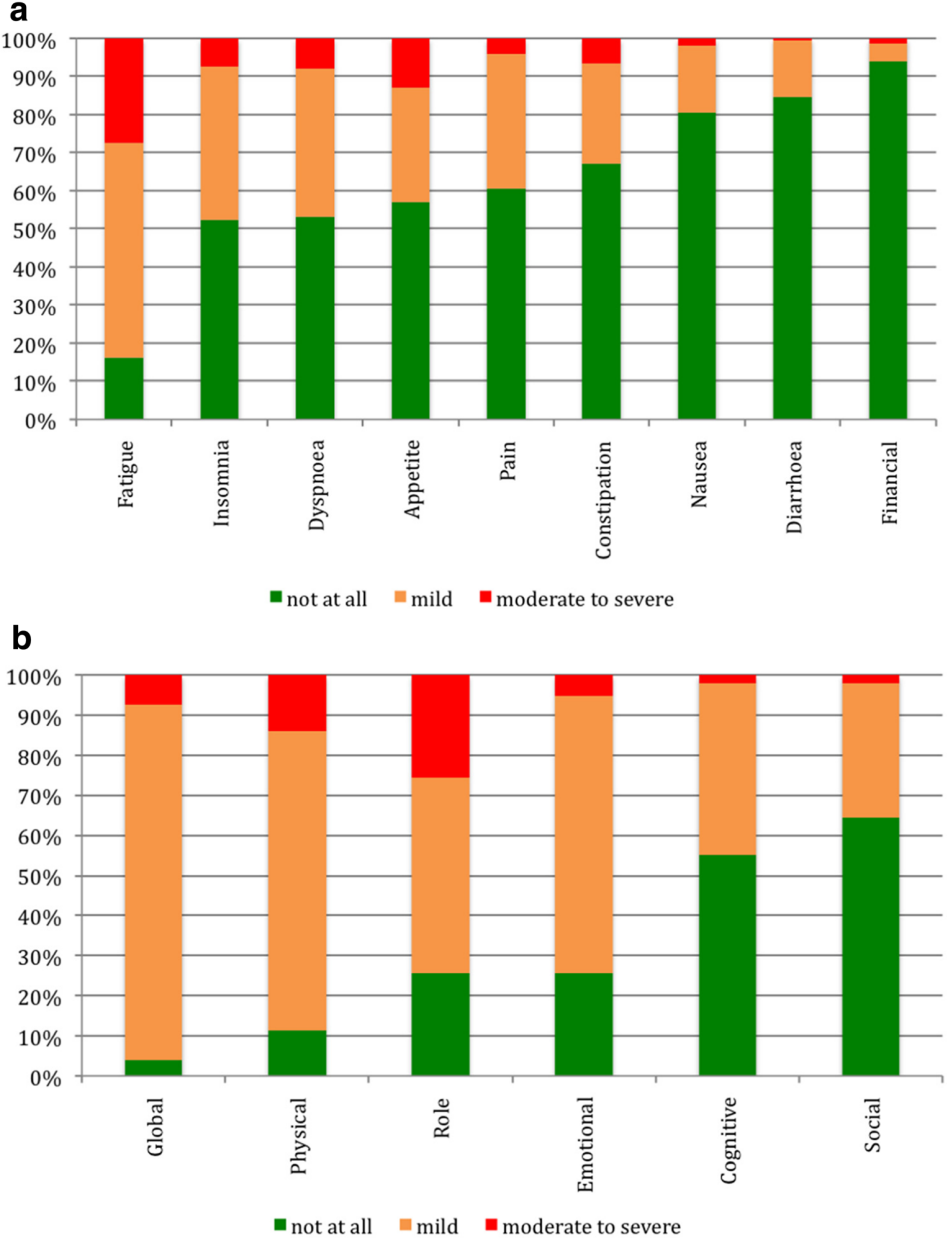
**a** Prevalence of symptoms as defined by EORTC QLQ-C30. **b** Prevalence of functional impairments as defined by EORTC QLQ-C30

As assessed by EORTC QLQ-C30, fatigue was the most frequently observed symptom (84%), with 56% of the study patients suffering from mild and 28% from moderate to severe fatigue (Fig. 1a). Patients with MDS, aNHL, AML, or iNHL were characterized by a higher prevalence of fatigue (90, 88, 81, or 77%) as compared with MPN (60%) (difference statistically not significant). Other symptoms, namely insomnia, dyspnea, loss of appetite, pain, and constipation, were reported by about one-half to one-quarter of our patients. In contrast, the symptoms nausea, diarrhea, or financial problems were rarely observed (< 20%) (Fig. 1a). Functional impairments were observed in a relevant proportion of patients (Fig. 1b). Restrictions were most frequently detected in global and functional health. In detail, mild or moderate/severe restrictions were observed in global (89 and 7%) and in physical health (75 and 14%) and in emotional functioning (69 and 5%). Role functioning was mildly impaired in 49% and moderately/strongly impaired in 26%. More than half of the patients reported no cognitive or social restrictions, with less than 5% being severely impaired in these domains.

### Association between fatigue and geriatric impairments and inflammation markers

Fatigue revealed a pronounced inverse correlation with distinct functional impairments and symptoms defined by EORTC QLQ-C30. The highest correlation was observed between fatigue and role functioning (rho = − 0.729; *p* < 0.001) (Table 2). Similarly, physical functioning and global health status were characterized by a marked correlation with fatigue (rho = − 0.688 and − 0.516, respectively) (*p* < 0.001). Fatigue showed the strongest correlations with the following symptoms: insomnia (rho = 0.508), dyspnea (rho = 0.417), and loss of appetite (rho = 0.411) (*p* < 0.001). Remarkably, fatigue was significantly associated with geriatric impairments in MGA. In detail, manifest fatigue was highly significantly associated with impaired performance status (WHO and Karnofsky Index (rho = 0.586 and − 0.613, respectively)), impaired G8 screening (rho = − 0.458), and depression scoring (GDS; rho = 0.543) (*p* < 0.001). Moderate correlations were observed with functional capabilities (IADL5, ADL, IADL; rho = − 0.346, 0.312, and 0.2840, respectively; *p* < 0.001), objective functional capacities (TUG; rho = 0.323; *p* < 0.001), and comorbidities (CIRS-G total; rho = 0.359) (*p* < 0.001). Remarkably, pronounced fatigue was significantly associated with low serum albumin (rho = − 0.295; *p* < 0.001) and markers of inflammation such as CRP (rho = 0.246; *p* < 0.05) and fibrinogen (rho = 0.227; *p* < 0.05) (Table 2).

**Table 2:**
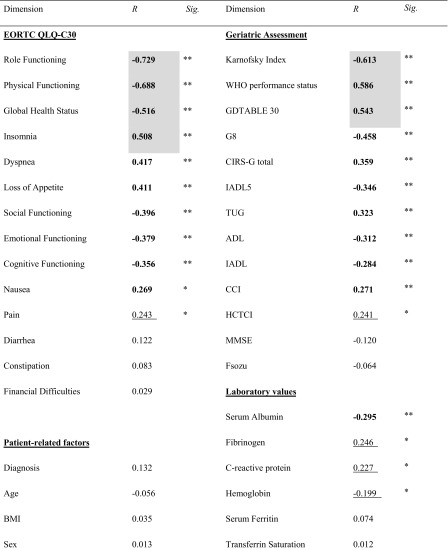
Association between fatigue and demographic data, geriatric impairments, and laboratory parameters

Correlation coefficients based on Spearman rho analysis are shown. High correlation coefficients (> 0.5) are bold and grey, moderate correlation coefficients (> 0.25) are bold, and weak correlation coefficients (> 0.15) are underlined. Statistically highly significant *P* < 0.001**; statistically significant *P* < 0.05*

*G8–G8* screening questionnaire, *BMI* body mass index, *ADL* activities of daily living, *IADL* instrumental activities of daily living, *GDS 30* geriatric depression scale 30, *Fsozu* Fragebogen zur sozialen Unterstützung, *MMSE* mini mental state examination, *CCI* Charlson comorbidity index, *HCTCI* hematologic cell transplantation comorbidity index, *CIRS* cumulative illness rating scale for geriatrics, *TUG* Timed Up and Go

### Impact of fatigue on clinical outcome

**Fig. 2:**
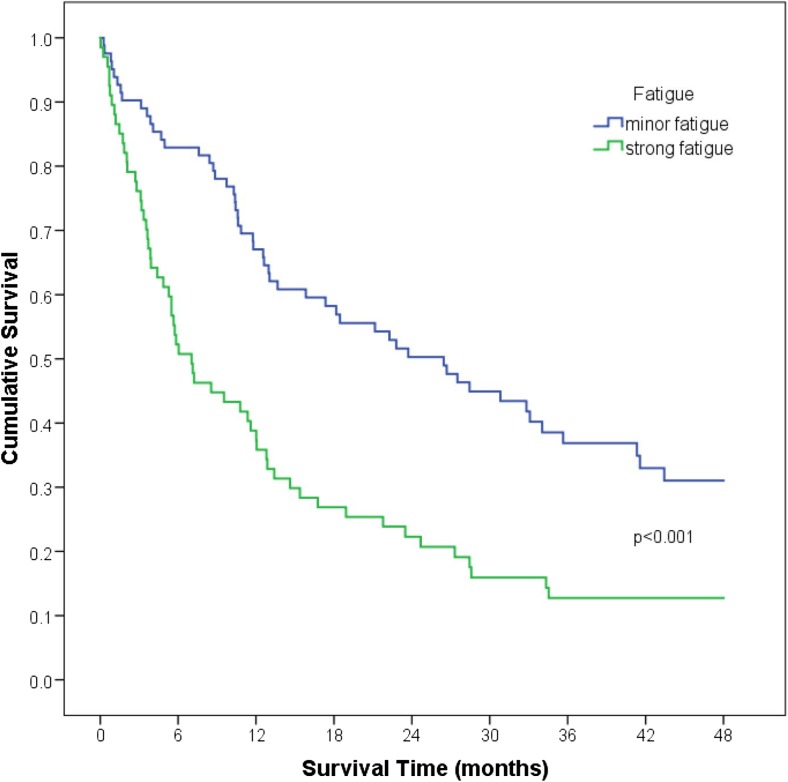
Pronounced fatigue is significantly associated with shortened overall survival at 4 years

Fatigue was highly significantly associated with unfavorable overall survival at 4 years, as assessed by Kaplan-Meier analyses. Median survival in the group suffering from no or mild fatigue was 26.4 months, while patients with a high fatigue burden had a median survival time of 7.0 months (*p* < 0.001) (Fig. 2). Univariate Cox regression analyses revealed a significant association between fatigue and increased mortality. Moreover, impairments in distinct domains of geriatric assessment including performance status, IADL, MMSE, GDS, F-Sozu, CCI, G8 scoring tool, polypharmacy, and loss of appetite as well as the number of impaired geriatric scores were significantly associated with unfavorable outcome. In addition, elevated serum ferritin levels and an AML diagnosis were negative predictors of survival (Table 3). All parameters significant in univariate analyses were included in multivariate analyses. Based on this approach, self-reported fatigue, the diagnosis of AML, and elevated serum ferritin levels remained independent significant predictors for survival (Table 3).

**Table 3:**
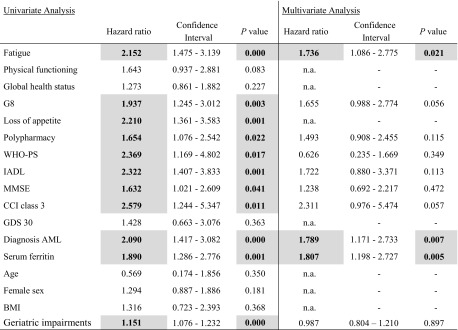
Prognostic parameters for overall survival

Significant values are displayed in gray

Definitions: fatigue—EORTC QLQ C30 fatigue > 45; physical functioning—EORTC QLQ C30 physical functioning > 66; global health status—EORTC QLQ C30 Global health status > 66; G8–G8 screening tool < 15; loss of appetite—G8 loss of appetite > 1; polypharmacy—> 3 medications; WHO PS—WHO performance status > 2; IADL—instrumental activities of daily living < 5; MMSE—MMSE < 25; CCI class 3—Charlson comorbidity index > 3; GDS 30—Geriatric Depression scale > 22.85; diagnosis AML—diagnosis acute myeloid leukemia; serum ferritin—serum ferritin > 400 μg/l; age—age > 80 years; sex—sex female; BMI—body mass index > 25 kg/m^2^; geriatric impairments—> 3 impairments in MGA

## Discussion

Hematological malignancies at advanced age are frequent and fatigue is a troublesome symptom in these patients. This is the first study to analyze fatigue, as defined by the EORTC-QLQ C30, prospectively in a well-defined cohort of older patients with various hematological malignancies at initial diagnosis. Importantly, fatigue is compared with geriatric impairments and with clinical outcome.

This study demonstrates troublesome symptoms and restrictions in HRQoL, namely in physical, role, and emotional functioning in a relevant proportion of patients with blood cancer. Remarkably, fatigue is the most frequent and distressing symptom, supporting observations in patients with MDS and multiple myeloma. As compared with high-risk MDS patients, our cohort was characterized by a slightly lower overall prevalence of fatigue (84 vs 92%, respectively) and less frequent moderate/severe symptoms, namely dyspnea, insomnia, and loss of appetite (< 13 vs > 20%, respectively) [6]. These differences might be explained by the more unfavorable clinical presentation and advanced disease of the MDS cohort, which contained exclusively high-risk patients. Similarly, patients with multiple myeloma were characterized by more pronounced fatigue in the cohort of Strasser-Weipl [19] (mean 49.1) and in the Nordic Myeloma Study Group [20] (mean 51.9) as compared to this analysis (mean 45.6). This observation may be explained by the advanced disease stage of myeloma patients, all of whom needed therapy [19, 20]. A prospective Danish study from a comprehensive cancer center showed more moderate/severe fatigue (61%), loss of appetite (55%), and impairments in role functioning (70%) than did our results [21]. The higher prevalence of severe impairments might be based on a possible selection bias, as Danish patients were hospitalized and both hematological (*n* = 52) and solid tumors (*n* = 72) were included. Thus, direct comparisons with data from the literature are flawed by the fact that most studies were not performed at initial diagnosis or used different selection criteria. A Danish cohort of 470 patients with malignant hemopathies revealed lower overall (55 vs. 84%, respectively) and less pronounced (moderate and severe fatigue 20 vs. 28%, respectively) fatigue than did this study [16]. This difference might be explained by the smaller proportion of aggressive entities such as AML in the Danish cohort (7 vs 32%, respectively) and the inclusion of patients at different stages of their disease trajectory. Thus, in many patients (38%), time from diagnosis was more than 5 years, which may suggest a selection bias in favor of long-term survivors.

An essential finding of this study is the correlation between pronounced fatigue and overall symptom burden, namely insomnia, dyspnea, and loss of appetite. Patients with higher levels of fatigue also had a greater overall symptom burden. These findings demonstrate that patients with a hematological malignancy report a wide range of symptoms and support observations on the clustering of symptoms [22]. Moreover, prominent fatigue is associated with impaired subjective role and physical functioning and global health status. Thus, recognition of fatigue is recommended to better understand the effect of disease burden on QoL of patients and might help physicians improve symptom management as suggested by Efficace et al. [6]. Evaluation of fatigue may represent a predictive parameter for impairments in overall health as well as in physical, role, and social functioning [22]*.*

Prominent fatigue was significantly associated with geriatric impairments, namely reduced performance status as defined by the WHO and the Karnofsky Index, objective physical capacities in the Timed Up and Go test (TUG), geriatric depression scoring (GDS), and impaired G8 scoring at initial diagnosis. The latter score represents a geriatric screening tool that reflects to a large part the nutritional situation in a given patient, as three out of eight items address nutritional aspects [23]. Correlation between fatigue and nutritional status is supported by the correlation between fatigue and the symptom “loss of appetite” in EORTC QLQ-C30 and low serum albumin, which represents a surrogate serum parameter of malnutrition [24]. Thus, the correlation between increased fatigue and parameters of nutrition, as defined by different aspects such as self-reported, externally observed and laboratory evaluation is impressively underlined. Similarly, physical functioning as self-rated in EORTC QLQ-C30, as rater-observed in WHO and Karnofsky status, ADL, IADL and in objective functional assessment in TUG, was highly associated with elevated fatigue. These observations also strikingly support the pronounced relationship between physical activities from both the patient’s and the physician’s perspective and fatigue.

Our observations extend data from the literature, which have demonstrated a strong correlation between fatigue and depression and decreased performance status [25–28]. However, these analyses were restricted to cohorts of hematological and solid tumors [25] or solid tumors [27, 28] and were not performed at initial diagnosis, but at later time points in the course of the disease [26–28]. Thus, our observations are relevant as they give evidence for an association between fatigue and geriatric restrictions in hematological malignancies at initial diagnosis. These findings might identify symptoms and restrictions, which might reflect the so-called fatigue syndrome. This may ultimately result in the frailty phenotype, which is characterized by impaired functional capacity and decreased mood [29]. Understanding these mechanisms may be clinically relevant, as frailty has been associated with poor therapeutic response, increased toxicity, and poorer survival for patients with hematological malignancies [30].

Comparison with laboratory parameters reveals a weak but significant correlation between fatigue and markers of inflammation. Thus, the concept of an underlying inflammatory process in aging and compromised health status is underlined and may form the basis for individualized approaches in future [31]. The weak and incomplete correlation between hemoglobin levels and fatigue from the literature is supported [32]. Thus, anemia is a weak determinant of reduced HRQoL, and other factors may be more relevant to determine hemoglobin levels [8].

An important observation in this analysis is that self-reported fatigue provides independent prognostic information for survival in elderly patients with hematological malignancies, even after adjustment for demographic and clinical factors. These observations are in line with evidence demonstrating the prognostic relevance of PROs: self-reported health has been shown to be among the strongest predictors of survival in a UK population-based study [33]. Similarly, the relevance of PROs including HRQoL for prognostication has been described in different cancer subtypes [34, 35] and in IPSS Int-2 and high-risk MDS [36]. Our findings complement these data and establish HRQoL as a relevant prognostic factor in prospective studies in hematological malignancies. These findings have important implications for clinical practice and the design of clinical endpoints. Based on this evidence, fatigue might be considered a standard stratification factor in future [34]. Moreover, prediction of clinical outcome might be improved by the combination of disease- and patient-related factors resulting in an integrated prognostic model as recently demonstrated in MDS [17].

To explain the association between fatigue and unfavorable clinical outcome, different pathophysiological mechanisms have been discussed. Inflammatory cytokine dysregulation has often been correlated with fatigue in patients with cancer [37]. The reason why patients with hematological malignancies, who have only mild anemia, nevertheless show severe symptoms of fatigue is unclear, but this fatigue has been postulated to result from disease-associated inflammatory changes that contribute to impaired QoL [31]. Molecular aberrations have been postulated to expand the malignant clone and may in addition contribute to inflammatory processes [38]. However, the underlying mechanisms of fatigue are far from being understood and deserve further analysis. Besides fatigue, serum ferritin has been demonstrated to be an independent prognostic parameter in this study. As serum ferritin reflects not only iron overload but also serves as an acute-phase reactant, the relevance of inflammation for the prognosis of patients might be underlined [39].

The strengths of our study are a parallel patient evaluation at initial diagnosis, a relatively long follow-up period, and a cohort consisting of elderly patients with various hematological malignancies. Thus, conclusions may be generalized for different subtypes of blood cancer. Limitations that should be taken into account are small patient counts for some entities, no repeated application of questionnaires, and thus no longitudinal data on fatigue. Future analyses will focus on distinct patient subgroups and on follow-up data.

## Conclusion

We observed a high prevalence of fatigue in older patients with a hematological malignancy. Pronounced fatigue was associated with impairments in role and physical functioning, performance status, and impaired depression scoring. Fatigue was identified as an independent adverse factor for overall survival. This study highlights the need for structured evaluation of fatigue and for treatment interventions that decrease fatigue and may thus positively affect clinical outcome.
